# Modification of the Interfacial Interaction between Carbon Fiber and Epoxy with Carbon Hybrid Materials

**DOI:** 10.3390/nano6050089

**Published:** 2016-05-12

**Authors:** Kejing Yu, Menglei Wang, Junqing Wu, Kun Qian, Jie Sun, Xuefeng Lu

**Affiliations:** Key Laboratory of Eco-textiles, Ministry of Education, Jiangnan University, Wuxi, Jiangsu 214122, China; melody1875155@163.com (M.W); wjq522@126.com (J.W); qiankun_8@163.com (K.Q); sunjie@jiangnan.edu.cn (J.S); sandylxf@tom.com (X.L)

**Keywords:** carbon nanomaterials, interfacial interaction, carbon fiber, epoxy

## Abstract

The mechanical properties of the hybrid materials and epoxy and carbon fiber (CF) composites were improved significantly as compared to the CF composites made from unmodified epoxy. The reasons could be attributed to the strong interfacial interaction between the CF and the epoxy composites for the existence of carbon nanomaterials. The microstructure and dispersion of carbon nanomaterials were characterized by transmission electron microscopy (TEM) and optical microscopy (OM). The results showed that the dispersion of the hybrid materials in the polymer was superior to other carbon nanomaterials. The high viscosity and shear stress characterized by a rheometer and the high interfacial friction and damping behavior characterized by dynamic mechanical analysis (DMA) indicated that the strong interfacial interaction was greatly improved between fibers and epoxy composites. Remarkably, the tensile tests presented that the CF composites with hybrid materials and epoxy composites have a better reinforcing and toughening effect on CF, which further verified the strong interfacial interaction between epoxy and CF for special structural hybrid materials.

## 1. Introduction

Fiber-reinforced polymers (FRPs), as a type of new engineering material, have been increasingly considered, owing to their outstanding mechanical properties and low density [1,2,3]. FRPs have promising applications in the automotive, aerospace, and construction fields, among others [4,5]. The mechanical properties are largely governed by the interfacial interaction between fibers and polymers. However, the interfacial interaction is also the weakest part in the composites [6], which can be attributed to the different mechanical properties between matrices and fibers because of the formation of an interphase region in the matrix close to the surface of the fibers [7]. Lee *et al.* [8] studied the effects of lysine-based diisocyanate (LDI) on the properties of biocomposite. LDI works as a coupling agent and the biocomposite is made from poly (lactic acid) (PLA), poly (butylene succinate) (PBS), and bamboo fiber (BF). The result indicated that both PLA/BF and PBS/BF composites yield better tensile properties because of the improved interfacial adhesion. Matuana *et al.* [9] suggested that the surface properties at the interface between the thermoplastic and the cellulosic fibers strongly influence the mechanical properties of the plastic/cellulosic fiber composites. They treated fibers with γ-aminopropyltriethoxysilane (A-1100), dichlorodiethylsilane, phthalic anhydride, and maleated polypropylene to improve the interfacial adhesion between fibers and plastic. Tang *et al.* [10] reviewed the methods for improving the interfacial adhesion between carbon fibers (CFs) and a polymer matrix. They focused on the surface physico-chemistry of fibers, including its surface chemical groups and microstructure, morphology, surface area, and surface free energy. Different kinds of nanoparticles were used to modify the fiber and epoxy composites to enhance mechanical properties. Yu *et al.* [2] enhanced the interfacial bonding of the CF and epoxy composites by using the multi-walled carbon nanotubes (MWCNTs) and silane coating. The results showed that the interfacial shear strength increased significantly. Park *et al.* [11] introduced a novel layer-by-layer assembly for the surface modification of glass fibers to enhance the interfacial properties between glass fibers and the epoxy matrix. They found that the surface free energy and the interfacial shear strength of the glass fibers uniformly coated with graphene oxide (GO) and aramid nanofiber (ANF) multilayers were improved by 23.6% and 39.2%, respectively.

In recent years, carbon nanomaterials, especial for MWCNTs [12] and grapheme [13], have been widely used to enhance the interfacial interaction for their unique properties, including physical strength [14] and chemical stability. Unfortunately, the dispersion of the carbon materials in polymers is a significant problem that limits their use in various fields because of the strong van der Waals force between MWCNTs and grapheme [15]. Hence, in order to improve the dispersion and the mechanical properties, hybrid materials of MWCNTs and graphene nanoplatelets (GnPs) appear because of their synergistic effect and special structure. The literature shows that the hybrid materials can be successfully prepared with different kinds of methods, such as the chemical vapor deposition (CVD) method [16,17] and solution mixing [18].

In our previous study [19,20], the MWCNTs and GnPs hybrid materials were successfully prepared with different feasible methods, which could combine the advantages of MWCNTs and GnPs to undertake a more external load. The special three-dimensional structure could make them disperse well in the epoxy matrix. In this work, we planned to use the MWCNTs and GnPs hybrid materials to enhance the interfacial interaction between CFs and the epoxy matrix. The epoxy resin composites with hybrid materials have excellent mechanical properties for the strong chemical bonding between the hybrid materials and the epoxy matrix. Moreover, the good dispersion of the hybrid materials in the epoxy also contributed to a strong interfacial interaction. In order to improve the interfacial interaction between the CF and the epoxy, we used the epoxy composites with the carbon nanomaterials to modify the CF. The nanoparticles act as a “buffer” to bear external load and avoid stress concentration. Thus, the homogeneous dispersion of hybrid materials in epoxy matrix is expected to provide the excellent mechanical properties of fillers/epoxy/CF composites.

## 2. Materials and Methods

Hydroxyl multi-walled carbon nanotubes (MWCNTs-OH, 95% pure, length of <5 µm, main range of outer diameter was 20 to 40 nm) were purchased from Shenzhen Nanotech Port Co Ltd. (Shenzhen, China). Hydroxyl graphene nanoplatelets (GnPs-OH, diameter of 1 to 20 µm, thickness of 5 to 15 nm) were purchased from Xiamen Knano Graphene Technology Co. Ltd. (Xiamen, China). Acryloyl chloride was supplied by J & K Scientific Ltd. (Shanghai, China). Tetrahydrofuran (THF), 1,4-dioxane and 2,2′-azosiobutyrontrile (AIBN) were purchased from Sinopharm Chemical Reagent Co. Ltd. (Shanghai, China). Epoxy resin (EP, hexahydrohsphenol-A diglycidyl ether), curing agent (methylhexahydrophthalic anhydride, 98 wt %), and accelerant (2-Ethyl-4-methylimidazole, 96 wt %) were supplied by the Suzhou Dongwu Glass Instrument Co., LTD. Spread CF tow (T700, 12K) was supplied by Toray Industries (Nantong, China).

### 2.1. Preparation of Fillers/Epoxy/Carbon Fiber Composites

The fillers and epoxy and CF composites were prepared according to the national standard GB/T3362-2005. Firstly, we prepared the pure epoxy composite system by adding epoxy resin, curing agent, and catalyst into a breaker (the weight proportion of epoxy resin: curing agent: catalyst is 100:70:1 [21]). Secondly, the GnPs-OH, MWCNTs-OH, and the hybrid materials were added into the pure epoxy composite (unmodified epoxy composites) system, respectively with 0.3 wt % concentration. The mixture process for the epoxy composite was developed with a mechanical mixer (JJ-1300W, Phil Pratt Experiment Instrument Factory of Changzhou, Changzhou, China). Then, the epoxy composites system was treated via ultrasonic and stirring for 6 h at room temperature to make fillers disperse evenly in the pure epoxy. Finally, the CFs were immersed in each epoxy composite system by hand-lay-up at room temperature for 3 min. The CF composite samples were prepared according to the following curing process without the external pressure: 80 °C 1 h, 120 °C 1 h, and 150 °C 1 h.

### 2.2. Characterizations

The morphologies of carbon nanomaterials were observed via transmission electron microscopy (TEM, Hitachi H-800-1; Hitachi Ltd. (Tokyo, Japan)), with an accelerating voltage of 20 to 30 kV. The dispersion state of different epoxy composites was observed by VH-S30 KEYENCE ultra-depth optical microscope (OM) (Shanghai, China). The curing reaction of epoxy composites was carried out by the DSC-Q200 differential scanning calorimeter (DSC, DSC-Q200; TA instruments (New Castle, PA, USA)) with the temperature control procedure: The samples were implemented at a heating speed 10 °C/min from 30–230 °C. Then, the temperature was reduced to 30 °C from 230 °C based on the speed of 30 °C/min and the temperature was then increased to 200 °C at a speed of 10 °C/min. Additionally, N_2_ gas at 50 mL/min was used in the DSC experiment. Rheological measurements were performed at room temperature using a stress-controlled rheometer (Rheometrics, Anton-Paar Physica MCR 301, Graz, Austria). The viscosity of the different epoxy composites with different nanoparticle and resin-hardener-accelerator systems were measured with a steady-state flow program, with the shear rate ranging from 0.01 to 100 s^−1^. The dynamic mechanical properties were tested via dynamic mechanical analyses (DMA, Q800; TA instruments (USA)). All sample dimensions were 60 mm × 15 mm × 2 mm and were carried out in the torsion rectangular mode with a constant frequency of 1 Hz and a heating rate of 2 °C/min in a temperature range of 30–200 °C. The tensile properties of composites were tested with an Instron3385H tensile tester (Phoenix, AZ, USA) with a 2-mm/min tensile speed. The effective length of all samples is 15 mm according to the GB/T1146-2005. The micrography of fracture surface of CF composites was observed via scanning electron microscopy (SEM, Hitachi SU1510; Hitachi Ltd. (China), Beijing, China).

## 3. Results and Discussions

### 3.1. The Microstructure Characterization of Carbon Hybrid Dispersions

**Figure 1:**
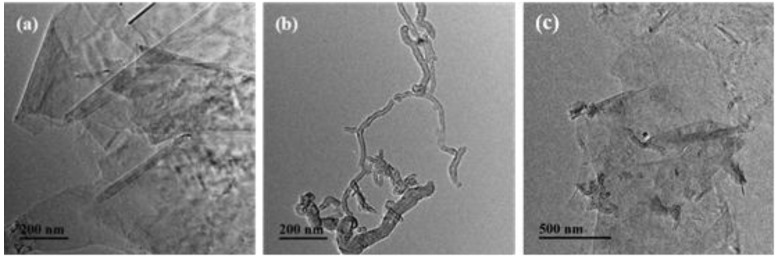
The transmission electron microscopy (TEM) images of (**a**) hydroxyl graphene nanoplatelets (GnPs-OH); (**b**) hydroxyl multi-walled carbon nanotubes (MWCNTs-OH); (**c**) hybrid materials.

Figure 1 present the microstructure of different carbon nanomaterials via the transmission electron microscopy (TEM). The obvious layers of GnPs-OH are clearly shown in Figure 1a. MWCNTs-OH are not tangled with each other, clearly shown in Figure 1b. The hybrid materials are shown in Figure 1c, the well-distributed MWCNTs-OH insert homogeneously in the layer of GnPs-OH. Moreover, the semitransparent substance (polypropylene acyl chloride (PACl)) can be clearly observed on the surface of the GnPs-OH and MWCNTs-OH to wrap the GnPs-OH and MWCNTs-OH. Combined with our previous study [19], it can be concluded that the MWCNTs-OH are grafted on the surface of GnPs-OH successfully by the chemical bond between hydroxyl groups of GnPs-OH or MWCNTs-OH and the acyl chloride groups of the bridge (PACl).

**Figure 2:**
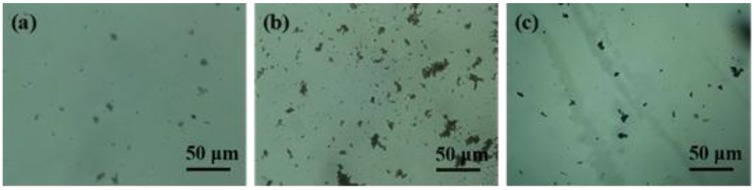
Optical microscope images of tetrahydrofuran (THF) suspensions with (**a**) GnPs-OH; (**b**) MWCNTs-OH; (**c**) hybrid materials at a filler concentration of 0.1 mg/mL.

The dispersion state of various fillers in tetrahydrofuran (THF) is investigated by the images of the suspensions at a filler concentration of 0.1 mg/mL. The dispersion solutions were prepared via 72 h sonication and 1 h sedimentation. Then, the prepared solutions were placed on a microscope slide with a syringe to observe under the optical microscope. Figure 2 shows the optical microscopy images of carbon nanomaterials at 500 magnification. There is a trend toward re-agglomeration with the 1 h sedimentation in the suspension. It can be seen that the GnPs-OH (a) homogenously distribute in the suspension, which may be attributed to the interaction between the layers of GnPs-OH from Figure 2a. It is clear that MWCNTs-OH (Figure 2b) show a severe aggregate phenomenon. The forming of aggregates is ascribed to the micrograph of hybrid material suspension. Compared to the GnPs-OH and MWCNTs-OH, the hybrid material suspension shows more homogenously dispersed aggregates and a smaller cluster size. Therefore, Figure 2 provides more evidence to prove that the dispersion of hybrid materials is superior to the GnPs-OH and MWCNTs-OH. In the hybrid materials system, with the 1D MWCNTs bridging adjacent 2D GnPs, GnPs may provide steric and electrostatic stabilization, preventing CNT re-agglomeration for their large surface area and space hindrance. Therefore, the hybrid filler is more prone to form a loosely packed 3D nanoparticle network in the solvent [15]. Moreover, the GnPs-OH and MWCNTs-OH are linked by the bridge (PACl, polypropylene acyl chloride), and the bridge could inhibit aggregation of GnPs-OH and MWCNTs-OH.

### 3.2. Differential Scanning Calorimetry (DSC) of Epoxy Composites

**Figure 3:**
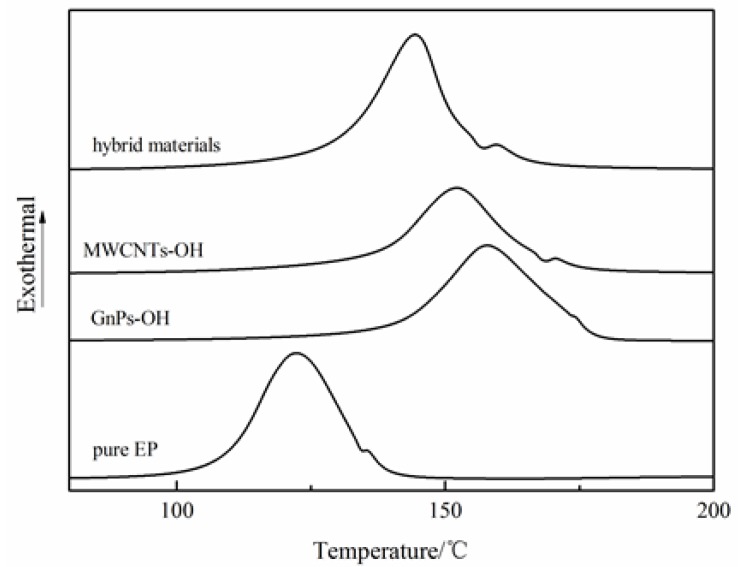
The curing reaction curves of pure epoxy and epoxy composites with different fillers. EP: pure epoxy.

**Figure 4:**
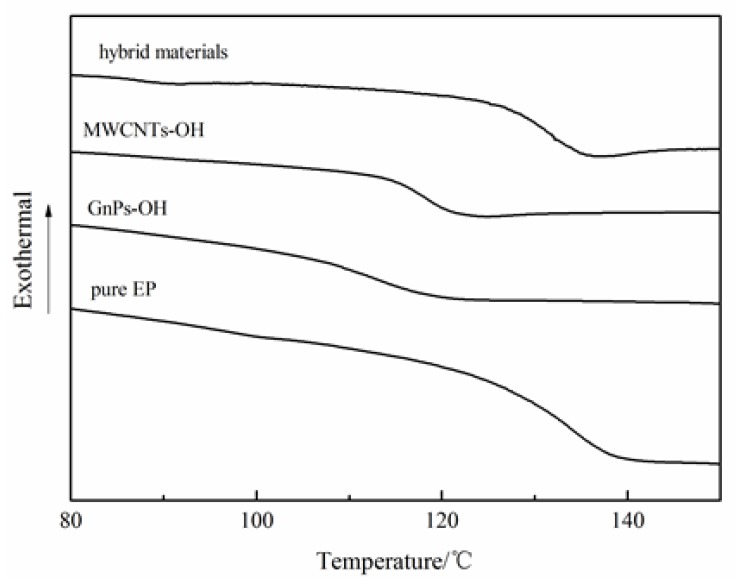
The glass transition temperature (*T_g_*) curves of pure epoxy and epoxy composites with different fillers.

The gelation, curing, vitrification can be studied with DSC tests. The curing reaction curves and glass transition temperature (*T_g_*) curves of epoxy with different fillers are shown in Figure 3 and Figure 4, respectively. The exothermal peak represents the curing process, and the *T_g_* of epoxy composites are presented in Figure 3 and Figure 4, respectively. The curing reaction of the epoxy composites with different fillers started later than that of the pure epoxy (EP), and the *T_g_* of the epoxy composites was lower than that of the pure epoxy. However, compared to the epoxy composites with different nanoparticles, the curing reaction of the epoxy composites with the hybrid materials started earlier, and the *T_g_* was higher than that of the epoxy composites with MWCNTs-OH and GnPs-OH, respectively. The phenomenon can be explained as follows: For the liquid uncured thermosetting polymers, in the curing process, the mobility of the epoxy segments decreased, and the glass *T_g_* increased. Generally, when the *T_g_* becomes higher than the curing temperature, the reaction terminates due to the “frozen polymer chain” [22]. Compared to the pure epoxy composites without nanoparticles, the carbon fillers can be considered as the physical interlock points in the epoxy matrix that hinder the mobility of polymer chains and inhibit the curing reaction of epoxy. However, in the epoxy composites with nanoparticles, the hybrid materials with a 3D structure present increased the free volume between the nanoparticles and the polymer matrix, which thereby enhanced the mobility of the segments and favored the curing process. Meanwhile, the epoxy composite with nanoparticles samples showed a lower *T_g_* than that of the pure epoxy, due to the enlarged free volume arising from the interface between the fillers and the epoxy resin. The epoxy composites with hybrid materials were observed to have a higher *T_g_* than the epoxy composites with MWCNTs-OH or GnPs-OH, respectively. This can be explained by the fact that the interrupted cross-linking networks that form between the resin and the PACl wrapped on the hybrid materials can also lead to an increased *T_g_* [23].

### 3.3. Rheological Behaviors of Epoxy Composites

**Figure 5:**
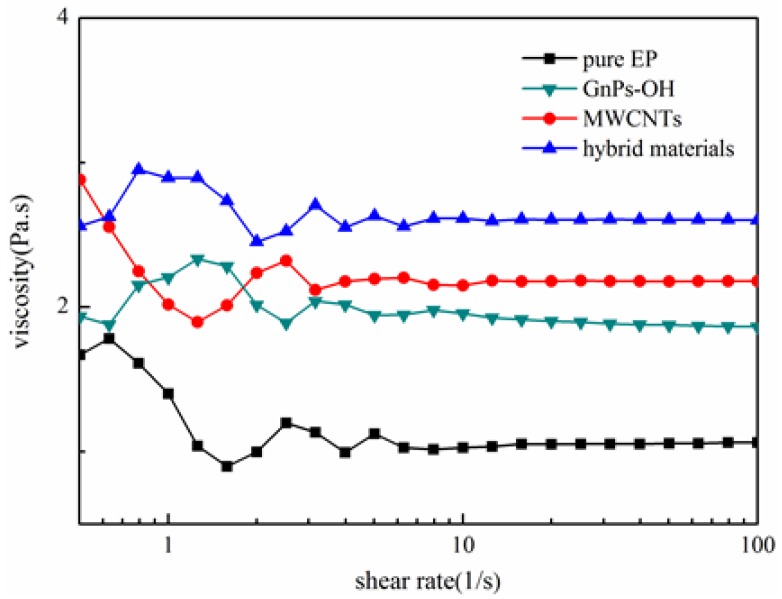
The viscosity *vs.* shear rate of epoxy and epoxy composites.

**Figure 6:**
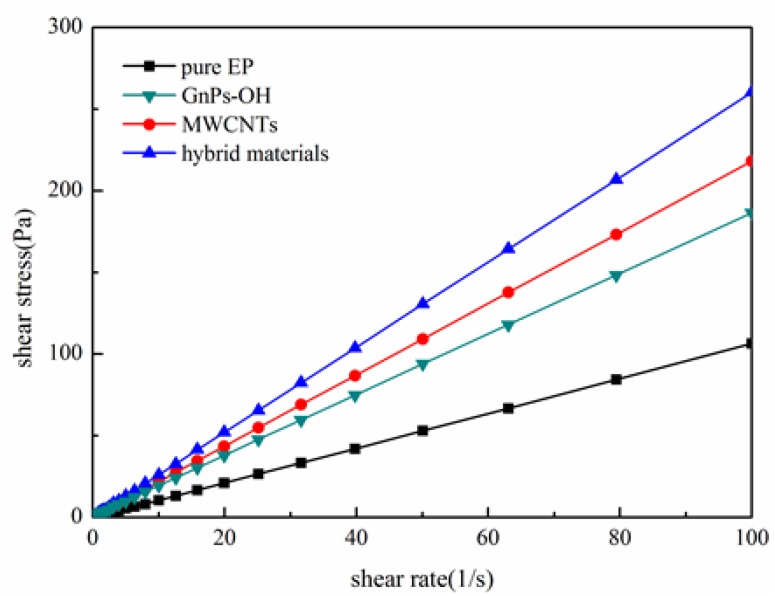
The shear stress *vs.* shear rate of epoxy and epoxy composites.

As shown in Figure 5 and Figure 6, the rheological behavior of the pure epoxy and epoxy composites are tested. The viscosity of the epoxy composites represent the dispersion of fillers in epoxy and the interaction between fillers and epoxy [24]. Figure 5 shows that the viscosity of epoxy and epoxy composites present an irregular curve trend at a low shear rate. With the increase of shear rate, the viscosity of epoxy composites tend to be a stable value. It is clear that epoxy composites with fillers exhibit a higher viscosity than the pure epoxy, and the epoxy composites with the hybrid materials show the highest viscosity. This phenomena suggest that fillers are well dispersed in the epoxy matrix, and the fillers and the epoxy matrix have better interface interaction, especially the hybrid materials. It is clearly that an increase in shear stress is observed with increasing shear rates in Figure 6. It is worth noticing that the shear stress of the epoxy composites with the hybrid materials has the highest shear stress, while the pure epoxy has the lowest shear stress at the same shear rate, owing to the improved interfacial action. The rougher surface of hybrid materials caused an increased interfacial friction for its special 3D structural, which in turn caused the increment of viscosity and shear stress.

### 3.4. Dynamic Mechanical Thermal Analysis (DMA) of Epoxy Composites

**Figure 7:**
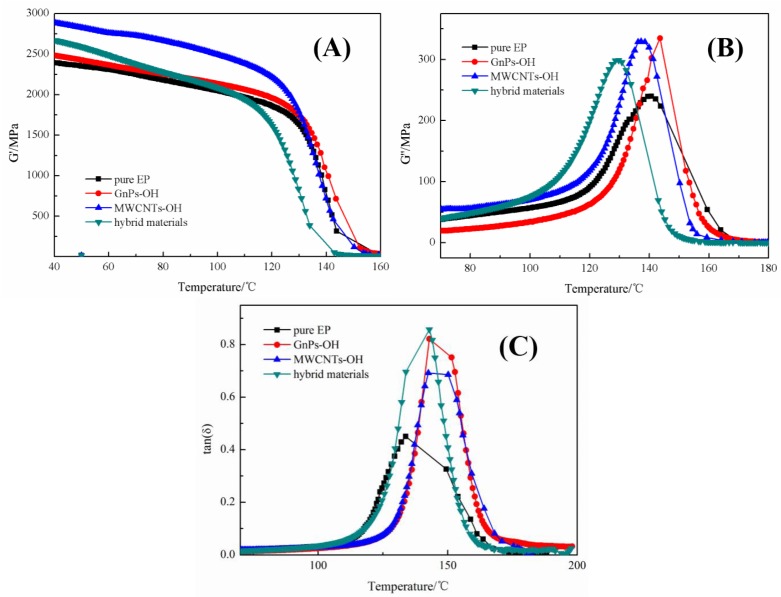
The dynamic mechanical thermal analysis (DMA) of (**A**) storage modulus (G′); (**B**) loss modulus (G″) and (**C**) dissipation factor (tanδ) *vs.* temperature.

Dynamic mechanical thermal analysis (DMA) shows the information on the storage modulus (G′), loss modulus (G″), and dissipation factor (tanδ) in the test temperature range. The elastic property and the energy storage of the epoxy composites can be characterized by the storage modulus. The viscous behavior and the energy dissipation of epoxy composites are represented by the loss modulus [25]. As shown in Figure 7A, the epoxy composites present a higher G′ than the pure epoxy at 40 °C and the G′ of composites with the hybrid materials is slightly lower than the highest value composites with MWCNTs-OH. The significant increase in G′ is ascribed to the strong interfacial interaction between fillers and epoxy matrix, which would reduce the mobility of polymer chains in the epoxy matrix [26]. The higher G′′ of epoxy composites with fillers in Figure 7B at 140 °C is related to the interfacial friction. With the increase in the temperature to the glass transition range (80–140 °C), significant changes could be observed in both G′ and G″ of the pure epoxy and epoxy composites. Both moduli of the composites became lower than those of the pure epoxy at 150 °C. The decreased G′ is attributed to the obstruction of fillers on the formation of the high cross-linked molecular structure of epoxy. The decreased G″ is due to the enlarged free volume between the fillers and epoxy matrix, which coincided with DSC results [23]. The damping behavior could be characterized by the tanδ. The higher the tanδ value is, the stronger the damping behavior of the system will be. As shown in Figure 7C, the tanδ of the epoxy composites with fillers is higher than the pure epoxy, and the epoxy composites with hybrid materials show the highest tanδ value. The tanδ value shows that the epoxy composites with hybrid materials had greater damping behavior, which led to the greater interfacial friction and interfacial interaction.

### 3.5. Mechanical Property of Carbon Fiber Composites

**Figure 8:**
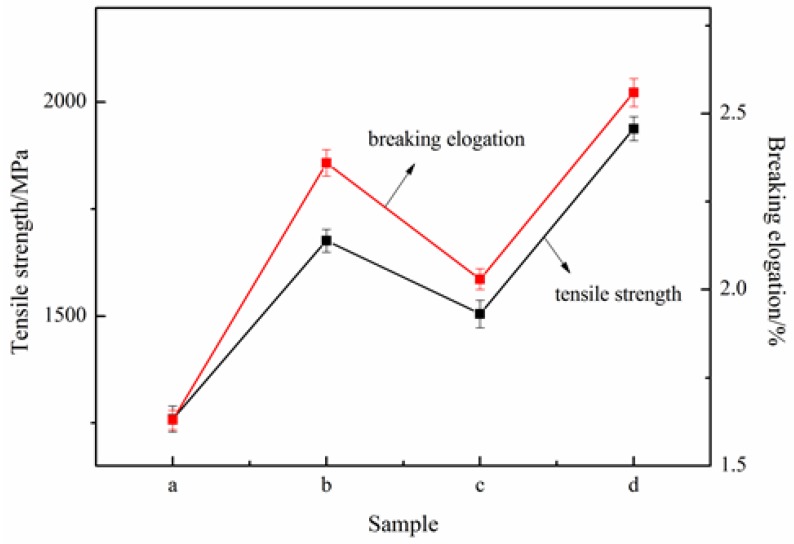
The tensile properties comparison of carbon fiber (CF) and epoxy composites (**a**) epoxy resin (EP)/CF; (**b**) GnPs-OH/EP/CF; (**c**) MWCNTs-OH/EP/CF; (**d**) hybrid materials/EP/CF.

**Figure 9:**
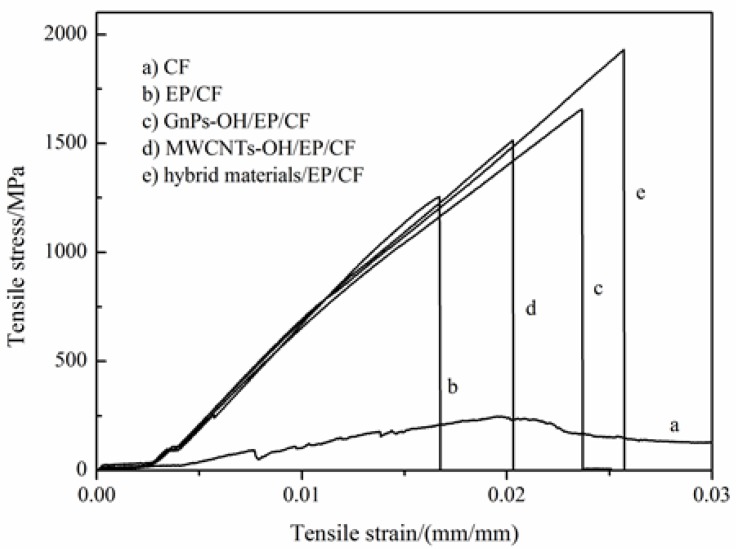
The tensile properties of CF composites.

The strength can be represented by the tensile strength, and the toughness often characterized by breaking elongation and integral area of tensile stress *vs.* strain curves. Figure 8 shows the tensile error bar for CF composites. It is clear that the error of tensile strength and elongation breaking of CF modified by different epoxy composites are within the margin of error. Combined with Figure 9, the tensile strength and elongation breaking of CF composites both show an increment with the addition of carbon nanomaterials. The tensile strength of the modified CF is higher than the CF. The CF, modified by hybrid materials/EP composites, presents the greatest strength, at about 1937 MPa, which is 53.97% higher than CF composites modified by pure epoxy (1258 MPa). Moreover, the highest elongation breaking is also the CF composites modified by hybrid materials and EP, about 2.56%, which is 57.06% higher than CF composites modified by pure epoxy. Considering the curve integral area, the largest curve integral area is presented by the CF composites modified by the hybrid materials and EP [27]. Thus, we can draw the conclusion that EP composites with hybrid materials could benefit from GnPs-OH and MWCNTs-OH to improve the strength and toughness of CF dramatically. One possible reason is the strong interfacial interaction among fillers, epoxy resin, and CF. The external load on epoxy or CF could be partially dissipated and delayed by the fillers. Thus, the 3D structural hybrid materials had the greatest advantage in improving the strength and toughness of CF.

**Figure 10:**
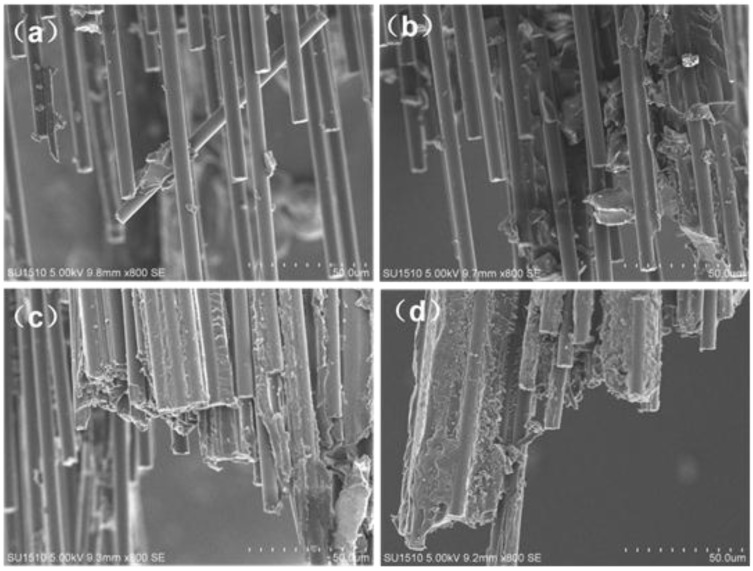
The SEM images of the fracture surface of CF composites (**a**) EP/CF; (**b**) MWCNTs-OH/EP/CF; (**c**) GnPs-OH/EP/CF; (**d**) hybrid materials/EP/CF.

Figure 10 shows the scanning electron microscopy (SEM) images of the fracture surface of the CF composites modified by EP composites. The fracture surface of fiber is smooth without massive epoxy, shows in Figure 10a (CF modified by pure EP). The fibers “pulled out” from the matrix represent the poor interface interaction between CFs and the epoxy matrix. The fiber surface attaches small MWCNTs-OH and epoxy composites shown in Figure 10b, while Figure 10c (CF, GnPs-OH, and epoxy composites) shows more attachment. As for the CF modified by hybrid materials/epoxy composites (Figure 10d), the CF are wrapped with the resin composites; they would not separate from one another when the external mechanical forces added. The phenomenon proves the strong interface interaction between the fibers, and the hybrid materials and epoxy composite. The good interface interaction is actually due to the hybrid materials distributed in the epoxy composites homogeneously. These hybrid nanoparticles with a stable three-dimensional structure are proposed to be a “buffer” that could bear external load and avoid stress concentration in the stretching process, while glomerate MWCNTs may lead to a concentration of stress.

## 4. Conclusions

The MWCNT and GnP hybrid materials perform a good dispersion in THF according to the TEM images and micrograph, whose 3D structure and polymer bridge inhibit the aggregation of GnPs-OH and MWCNTs-OH. For the nanomaterials/epoxy composites, the DSC results show that fillers could inhibit the mobility of polymer chains and strengthen the interfacial interaction in turn. The rheological behavior of epoxy composites indicates that higher viscosity and higher shear stress also provide evidence for strong interfacial interaction between fillers and epoxy matrices. Similarly, the interfacial friction, damping behavior, and free volume are obtained from DMA to characterize interfacial interaction. For the nanomaterials, epoxy, and CF composites, the CF modified by hybrid materials and EP performs the highest tensile strength and elongation breaking, benefitting from the 3D structural hybrid materials. According to the SEM photos of the fracture surface of CF composites, the hybrid materials and CF and epoxy composites attach with massive EP, owing to the hybrid materials distributed in the epoxy composites homogeneously, and these nanoparticles are proposed to be a “buffer” that can bear and spread external load in the stretching process.
